# European illegal puppy trade and organised crime

**DOI:** 10.1007/s12117-021-09429-8

**Published:** 2021-08-24

**Authors:** Jennifer Maher, Tanya Wyatt

**Affiliations:** 1 University of South Wales, Pontypridd, UK; 2 Northumbria University, Newcastle upon Tyne, UK

**Keywords:** Puppy trafficking, Illegal puppy trade, Organised crime, Transnational crime, Animal abuse

## Abstract

Organised crime groups’ involvement in illicit markets is a common focus of law enforcement and governments. Drug, weapon, human and wildlife trafficking (and others) are all illegal activities with link to organised crime. This paper explores the overlooked illicit market of puppies. We detail the state of knowledge about the organisation of the UK puppy trade, which includes irresponsible and illegal breeding of puppies throughout Europe and their often-illegal movement into the UK. In 2017, we conducted an analysis of hundreds of online advertisements in Scotland, 12 expert interviews, a stakeholder survey of 53 participants, and 40 focus groups across Great Britain. Our data suggest an organised illicit market running in parallel to the legal trade. We speculate as to whether at some point along the supply chain organised crime groups are responsible for the suffering and death of the puppies and the economic and emotional damage to ‘consumers’. Online monitoring and physical scrutiny at the ports must be improved to reduce non-human animal abuse. People buying puppies must also be made aware that their purchase could be profiting organised crime.

## Introduction

The pet trade is a lucrative commercial enterprise which involves moving thousands of species and millions of individual non-human animals^1^ across the globe. According to a Council of Europe report, half of European households own a pet of some kind, estimated to include almost 7000 different species. The reported value of industry turnover for those supplying pets and related goods is estimated to be several €10s of billions annually (Davenport and Colins 2016, p. 33). There is growing evidence that this trade has opened the door to criminality and is increasingly of interest to organised crime groups. Aspects of the better-known and larger illegal wildlife trade are facilitated by organised crime and the organised crime aspect is increasingly the focus of national and international regulation and enforcement (Arroyo-Quiroz and Wyatt 2019; UNODC 2020; Wyatt et al. 2020). Yet, while the illegal wildlife trade, including the trafficking of exotic pets and the potential role of organised crime, has been the focus of a significant amount of research in the last decade (see Arroyo-Quiroz and Wyatt (2019), Collard (2020), Sollund (2019), Van Uhm (2016), Wyatt et al. (2017a), Maher and Sollund (2016), among others), the trafficking of ‘domestic’ companion animals^2^ has received little attention.

This paper explores the emerging lucrative illicit market of puppies, which is largely overlooked in academic literature. According to FEDIAF (2020), more than 85 million households in the European Union had companion animals in 2019. Russia (not in the EU), Germany and the UK (then in the EU) are thought to have the most dogs (16.8, 10.1 and 9 million respectively) with high numbers also in Poland, France, Spain and Italy (7.7, 7.6, 7 and 6.7 million dogs) (FEDIAF 2020). This buoyant European commercial trade requires an unknown number of puppies to meet market demand, which is estimated in just 12 EU member states to be valued at €1.3 billion annually (IBF International Consulting et al. 2015). In the UK alone, there is an estimated annual sale of between 800,000 and 1.3 million puppies. Sales through advertising online are estimated to be worth approximately £130 million per annum (Maher and Wyatt 2019). Numerous reports (IBF International Consulting et al. 2015; IFAW 2012; Dogs Trust 2014; RSPCA 2012; Four Paws International 2013) have identified, a sea-change in the scale and nature of the puppy trade—including significant growth in large-scale industrial commercial breeders, international trade and the use of the internet as the medium for third-party sales. Consequently, alongside the organised and expanding legal trade is the development of an irresponsible and illegal trade in dogs, with growing evidence that it has attracted the attention of criminal networks across Europe.

In our UK research (Maher and Wyatt 2019; Wyatt et al. 2017b), we found puppy smuggling is lucrative due to high demand among consumers. Yet it carries little risk of detection, prosecution or punishment. Consumer behaviour, the preference for specific ‘designer’ dogs and implicit trust in online advertising, has greatly facilitated the illegal trade, as buyers become increasingly removed from the origins and movement of the dogs. Concurrently, we saw that offenders have developed fluid, organised and sophisticated strategies to reassure consumers in their purchases. Consumer demand is particularly relevant as the desire for dog companionship has apparently reached new highs during the ongoing period of isolation and working from home experienced in the COVID-19 pandemic (Kale 2020). Prior to this, puppy trafficking, as part of the European illegal pet trade was linked to serious and organised crime (European Parliament 2019) and argued to have become the third largest illegal trade in Europe (Eurogroup for Animals 2018). The COVID-19 driven market is likely to heighten criminal interest and involvement. A report based on the recent conference organised by Eurogroup for Animals (2020, p. 19) on behalf of the Croatian Presidency of the Council of the European Union argues “A change of attitude is needed in central and local government to accept that the puppy trade is controlled by criminal gangs in the same way as the drug trade or human trafficking are”. This potential link to organised crime is supported by Her Majesty’s Revenue and Customs [HMRC] whose dedicated puppy trade task force recovered over £5 million in unpaid taxes from 257 cases over a four-year period (HMRC and Stride 2019), though more information would be needed to firmly link this tax evasion to organised crime.

As mentioned, it is impossible to accurately quantify the scale of the European or UK puppy trade. Distinguishing between the legal, irresponsible and illegal trade is challenging for authorities and consumers. Estimates of the number and value of puppies responsibly, irresponsibly, or illegally bred and sold, or of active breeders and sellers and those who are acting unscrupulously, illegally or with organised crime groups are problematic. Part of the problem is knowing which aspects and actors are involved in organised crime. Thus, we begin by clarifying what constitutes organised crime groups, before sharing our findings on the structure and organisation of the UK puppy trade. There is a vast literature debating and theorising what organised crime is. We, too, have contributed to this discussion (Arroyo-Quiroz and Wyatt 2019; Wyatt et al. 2020). Without repeating those debates in this paper,^3^ we adopt a more straightforward approach to organised crime by employing the standardised definition of EUROPOL (2017). This is in part due to space, but also for practical reasons, in that we hope our findings can be useful to law enforcement agencies across Europe using the conceptualisation and definition of EUROPOL when evaluating the organised nature of puppy trafficking.

EUROPOL specifically departs from the UN Convention on Transnational Organised Crime’s (UNTOC) definition of organised crime (a group of three or more persons existing over a period of time acting in concert with the aim of committing crimes for financial or material benefit (UNODC 2000)) because “this definition does not adequately describe the complex and flexible nature of modern organised crime networks” (EUROPOL 2017: no page). EUROPOL’s (2017) analysis of organised crime groups [OCGs] has found a variety of structures (that is, hierarchies, loose temporary networks, small groups etc.) and a diversity of actors. Online activities are highly adaptive and lucrative and make up a substantial portion of black markets (EUROPOL 2017). Of particular relevance to our analysis of puppy smuggling is EUROPOL’s (2017: no page) observation that:

Many OCGs are highly flexible and able to shift from one criminal activity to another or to add new criminal activities to their crime portfolio. In many cases, OCGs operate on an on-demand basis and only become active once new profit opportunities emerge.

The integration of digital systems in many criminal activities and the expansion of the online trade in illicit goods and services is transforming serious and organised crime. Criminals are increasingly adapting the supply chain models of global online retailers.

We will return to this when assessing whether puppy trafficking is an organised crime.

Based on empirical research conducted in the UK in 2017, this paper evaluates the involvement of criminal organisations in the illegal puppy trade in the UK and possibly Europe. The research is situated within the distinct field of green criminology, a critical harms-based discourse, which focuses on exposing and challenging harms to animals and the environment (Beirne and South 2007; South and Brisman 2020). Green criminology, like organised crime research, is concerned with crimes of the powerful and crimes of great volume and (financial and welfare) cost. To ensure non-human animals are visible as victims (Beirne 1999), a broad definition of harm is adopted herein. The harms involved are viewed as serious crimes which deserve consideration alongside other serious and organised crimes. First, we briefly outline the methodology employed to conduct our data collection. We then provide an overview of the nature of the legal puppy trade in the UK gleaned from that research and literature^4^; in particular, we propose how the trade is structured and who the actors are along the supply chain. This is followed by details of the impact of the trade and of trafficking. We then detail the key research findings to identify the points along the supply chain and elements of the trade which are consistent with EUROPOL’s conceptualisation of OCGs. In particular, the behaviour of key stakeholders, such as the breeders, sellers, consumers and enforcement agencies are considered. We conclude by considering the implications associated with the involvement of OCGs in the illegal puppy trade and the responses to this element of the trade in the context of the wider political and economic climate.

## Methodology

In 2017, a mixed-methods research design, involving a systematic literature review and five stages of empirical data collection was adopted to determine the prevalence, nature and impact of the illegal puppy trade. While the initial project focused on Scotland, additional funding expanded the study to England and Wales. The literature reviewed included academic and grey literature from the UK, but also the wider European community and the US. Empirical data collection involved conducting an analysis of hundreds of online advertisements in Scotland, 12 expert interviews, a stakeholder survey of 53 participants, and 40 focus groups across Great Britain. From October 2016, all online advertisements for puppies in Scotland in seven key websites (Craigslist, Dogs & Puppies UK, Epupz, Freeads, Gumtree, Pets for Homes, and Pets Viva Street) were monitored for 12 weeks. Where possible, data was collected on the Local Authority (LA) location, breed, number per litter and sex of the puppies, price per puppy and in total, phone number, name of the seller, Kennel Club and LA registration, and any other information. To further understand the economics of the puppy trade, the Animal and Plant Health Agency (APHA) TRACES database was analysed. The database records the legal movement of dogs for commercial purposes (including rescues) and identifies transgressions within this trade. Trading Standards Scotland data details recorded complaints from puppy purchasers.

The semi-structured expert interviews included people with specialist knowledge and/or direct experience in responding to the puppy trade (i.e. NGOs, veterinarians, government employees and breeding standards organisation—quotations from these interviews are coded as ‘EI’—expert interview and a number). This was supplemented by an online survey used to capture the experiences of 53 key stakeholders working in related fields (i.e. dog walkers, groomers, trainers, boarders, and veterinarians) in order to learn about their dealings with consumers and their puppies, who may have come from irresponsible or illegal sources. Drawing upon findings from the economic, online advertisement, expert interview and online survey data, consumer focus groups were conducted (25 face-to-face and 15 over the phone) with 160 people around Great Britain who were considering buying or had recently (within the last two years) bought a puppy. Data were analysed through SPSS and NVIVO.

In recognition of the existing data limitations, this multi-method triangulated research design was utilised to validate the findings and negate some of the limitations (a majority of which stem from the samples obtained). For example, the purposive expert sample was small and through the snowballing process identified a larger number of NGOs with experience in responding to the trade. Consequently, the findings should be read to represent the values and experiences of a small number of experts, with an emphasis on NGO viewpoints. Both the survey and the focus groups are limited by selection bias. It is likely that only those people concerned about dog welfare chose to participate; the focus group demographics, for example, were mostly women aged over thirty. While the focus groups involved consumers, who bought through ‘assured’ breeders, registered breeders, friends, ‘backroom’ breeders, online traders, and illegal traders, those who had inadvertently or purposefully bought puppies from irresponsible and/or illegal sources were underrepresented. Participation was voluntary and due to the sensitive and emotional nature of the topic, those who engaged with questionable actions or who experienced the death of their puppies were unlikely to participate. Despite these limitations, the data collected represent all the key stakeholders in the puppy trade and provide useful insights into whether or not the illegal trade involves OCGs.

## The nature of the puppy trade

First, we provide an overview of the scale and value of the puppy trade in the UK, highlighting the structure and actors along the supply chain and the impact of the trade.

### Scale and value

As mentioned, the scale of the puppy trade is difficult to estimate. And the illegal trade is particularly difficult to address due to its hidden nature, the inconsistent data collection and sharing, the multiple regulatory frameworks and agencies, and limited law enforcement capacity, very little of which is specialised. The two—legal and illegal—are also intertwined and difficult to untangle. Thus, when speaking of scale and value it is hard to distinguish between legal and illegal. For example, one interviewee indicated that when the Irish government trade database recorded approximately 3,000 puppies as being transported commercially to the UK, he was able to “directly follow over 52,000 pups to the UK every year and that was the ones we knew about” (Wyatt et al. 2017b). As stated, it is not clear which of these are legal and which are not. Another example comes from the Dogs Trust (2018). A Hungarian puppy dealer detailed the weekly exportation of approximately 400 puppies from just his hometown, suggesting a trade of 20,000 puppies a year from one village. With an average price of £1400 per puppy, the annual turnover is £28 million. In this case, the trade was considered illegal because of the lack of registration and welfare. RSPCA inspector Briggs, from the special operations unit, has argued that criminals are driven by immense profits, with in-demand ‘handbag’ dogs “bought in Eastern Europe for as little as £20 ($29) and sold for as much as £1200 ($1700) in the UK, allowing even small-scale operators to make thousands of pounds every week” (Parkinson 2016: no page). Even though the exact amount of illegal trade is unknown, the Biocrime project which documents the trade of animals across the Austrian-Italian border found 53% of pets traded across the border had no documentation (Eurogroup for Animals 2020). Thus, there is the possibility that the illegal portion of the puppy trade is as, if not more substantial, than the legal trade.

### Structure and actors

The puppy trade has numerous stages and within these stages there are a variety of activities undertaken by a diverse group of actors (see Table 1). Portions of the trade may be all, or a mixture of:

1. legal regulated trade (in compliance with the complex interplay of local level governance and international regulations on breeding, rearing, movement, trade, selling and the welfare of the dogs)
2. legal unregulated trade (below the threshold requiring domestic regulation)
3. illegal trade (non-compliant with international and national regulations).

**Table 1:** The structure of the puppy trade

Stage		Activities (legal and illegal)	Actors
1	Planning	• Choose dogs (breed, colour etc.) • Choose location • Obtain registration/licensing permits • Fraudulently obtained or no permits	• Breeders • LA officials • Third party sellers (e.g. on-demand orders) • Consumers (e.g. market trends)
2	Breeding	• Breeding bitches • Veterinarian checks • LA (or equivalent) compliance checks • Inappropriate housing/welfare/numbers	• Dogs • Breeders • Veterinarians • LA inspectors • NGOs
3	Rearing	• Rearing and socialise puppies • Microchipping • Veterinarian checks • LA (or equivalent) compliance checks • Inappropriate housing/welfare/numbers • Forged documentation	• Puppies and dogs • Breeders • Veterinarians • LA inspectors • NGOs
4	Transporting	• Transporting in vehicles nationally and internationally • Compliance with EU Balai Directive and Regulation (EU) No 576/2013 [EU Pet Travel Scheme—PETS^a^] • Non-compliance/smuggling • Border checks	• Puppies • Breeders • Transport companies/workers • Border agents • Traders • Smugglers • NGOs
5	Advertising	• Place adverts on websites, social media and/or online classifieds • Non-compliance with registration/identification/microchipping details • Forged information	• Breeders • Traders • Internet and website companies • Consumers • LAs • NGOs
6	Selling	• Meet the puppies/mother • Deliver/pick up puppy • Exchange documents (passport, microchipping) • Payment • Report sales • Fraudulent paperwork and business • Fake houses and mothers • Non-compliance (e.g. with Lucy’s Law in England) • Illegal business • Tax evasion	• Puppies and dogs • Breeders • Traders • Consumers • Tax Officers • LAs • Consumer organisations (e.g. trade) • NGOs

The Balai Directive [Council Directive 92/65/EEC] and PETS [Regulation (EU) No 576/2013] regulate the movement of commercial and companion animals within the EU. New import/export requirements are being applied in a staged approach from 01 January 2021 as the UK has left the EU

The legal (both regulated and unregulated) and illegal trade of puppies are multi-stage processes. According to the data, those profiting from the puppy trade are a mixture of individuals selling occasional litters, hobby breeders, and small and large commercial enterprises. Across these groups, there are examples of good practice (such as complying with online advertising minimum standards, and vetting perspective buyers), evidence of unscrupulous breeders and traders (e.g. irresponsible breeding, rearing, and sales practices) and illegal activities (such as importing commercial dogs as pets, evading taxes, or using fraudulent pet passports). Experts described illegal actors as non-compliant individuals, organised crime groups and ancillaries (e.g. for transport). While it is not currently possible to identify the extent or specific roles occupied by OCGs in the trade, experts indicated actors in the illegal trade are becoming increasingly organised and sophisticated in their operation and existing crime groups are moving into the puppy trade. The diversity of actors adds to the difficulty in identifying the nature (and prevalence) of the legal and illegal trades. Those who are legitimate can easily become non-compliant, while those who purposefully smuggle and organise the illegal trade can use the legitimate trade to do so (e.g. laundering dogs).

To breed and sell puppies, actors must plan which dogs to buy and where to house them. Although regulations differ across the EU, if breeding enough dogs and acting legally, most likely the breeder must obtain a license or permit. Inspections by local authorities and veterinarians^5^ would also likely be involved at this stage (2 and 3 in Table 1). To commercially sell these puppies across the EU the regulations^6^ require the premises of origin to be a registered business. In the UK, licensed breeders are required to breed within the determined limitations of their establishment and welfare of individual mother dogs and provide a set standard of care and welfare. Non-compliance is evident in the illegal domestic trade, however, similar abuses among international breeding establishments is not necessarily illegal as there is no UK control over the breeding rates and conditions of puppies arriving from these establishments. The aforementioned IBF (2015) study found significant inconsistencies in licensing requirements and levels of compliance across EU member states. The possession of a licence or a registered business is no guarantee of legality or appropriate animal welfare standards. Our study respondents (i.e. experts, stakeholders and consumers) indicated limited enforcement resources resulted in poor licensing compliance and provided substantial opportunities for illegal trade. Further, in the UK, where it is possible to breed puppies unregulated (e.g. under the legal threshold of three litters a year) it is possible to trade illegally under the guise of unregulated legal trade.

To transport puppies internationally (Stage 4), either commercially or as companion animals, veterinarians must complete a health certificate (ITAHC) or a pet passport [PETS].^7^ Covert investigations by NGOs repeatedly identified many veterinarians in Eastern European countries who were paid to forge paperwork to facilitate the movement of puppies too young to legally travel. This research revealed inadequate and few checks at the borders, infamously evidenced by the ability to repeatedly import a stuffed, rather than a live, puppy into the UK (Dogs Trust 2014, 2018). During this stage of the (il)legal trade, transportation companies and their workers should encounter the puppies, dealers and persons transporting them. This may be airlines, but more commonly involves ferry companies or agencies operating tunnels and other border crossings (i.e. Eurotunnel between France and England). To avoid commercial checks, widespread abuse of PETS, implemented in 2012 to facilitate companion animals travelling with their owners across EU borders, was consistently identified by experts. As each person in a vehicle can travel with up to five companion animals, it has become a viable mechanism for illegal commercial sales. Furthermore, the movement of companion animals under PETS is not recorded, permitting offenders to make repeated journeys with different dogs without being identified. The increase in commercial imports to the UK, during COVID-19 restrictions on non-commercial movement across borders, supports this premise. While the movement of dogs to England and Wales under PETS declined by 88% in May (compared to previous years), the movement of commercial dogs, measured by Intra Trade Animal Health Certificates (ITAHC) (issued between March and October 2020) rose by 87% (on numbers for May to September 2019)—the highest number of ITAHCs issued monthly since records began (RSPCA 2020). Whereas 5 years ago most puppies were being transported in vans, it is now thought that cars, being less conspicuous, are increasingly being used to transport puppies internationally throughout Europe (Eurogroup for Animals 2020). Experts in our research identified a fluid network of offenders, who were swift to respond to enforcement changes.

Stage 5—advertising—may overlap with previous stages as puppies are advertised before being born, early in their lives, or when they are ready to be transported—creating an on-demand process. Advertisements may be placed from outside of the destination country of the puppies. For instance, we found that advertisements even with local addresses in Scotland could be for puppies bred out of the country, but being sold by local third-party people, who had not bred the puppies (Wyatt et al. 2017a, b). In response to UK awareness campaigns, local sellers used fake houses and female dogs to allow consumers to view puppies in their ‘home’ with their ‘mother’. That way during Stage 6—selling—consumers visiting perspective puppies do not suspect the puppy has come from out of the country and/or a puppy farm. Furthermore, the seller is not re-traceable due to their frequent movements from rented accommodation. While UK legislation has recently become more robust (e.g., Lucy’s Law), requiring all online advertisements from businesses to provide crucial information for traceability and prohibiting third-party sales, this is not adequately enforced, and the latter does not currently apply to international trade. Although the income from all regulated legal puppy sales should be reported to the relevant tax authority, the recent HMRC figures detailed above indicate considerable tax evasion.

### Impact and implications

The puppy trade requires the systematic exploitation of dogs. Within the UK regulated trade minimum standards of welfare and treatment can reduce some of these harms, while the harms experienced by dogs in the illegal and irresponsible trade are pervasive, diverse, and serious (Dogs Trust 2018; Eurogroup for Animals 2020). The impact begins at the point of origin and is evident at every stage in the trade, including post-purchase. Harms to the non-human animals include, but are not restricted to, inappropriate breeding (with exaggerated features or with inherited disorders that compromise welfare), health (no or inappropriate veterinary care, removal from mother too early, exposure to diseases, premature death), living (unsanitary, cramped and noisy housing, poor care, undernourishment, inability to express natural behaviours, no socialisation with other dogs or people) and transportation conditions (inappropriate housing, long journeys without access to food, water or breaks, without appropriate vaccinations to avoid disease transmission), and abandonment and euthanasia (due to poor compatibility with owners, long term health problems and/or behavioural problems).^8^ The (il)legal commercial exploitation of millions of non-human animals causes extensive and persistent suffering, with little recognition of animal sentience. This is despite extensive scientific basis evidencing they are sentient and can and do experience pain and pleasure. Within the illegal trade, animal welfare is frequently sacrificed as profits are likely, even when a large percentage of animals die pre- or post-sale, because of these conditions:

if you’ve got 40 … and three happen to die they’re just getting kicked out at a lay-by… if I pick up 100 and I only end up with 50 I’m still going to make a good profit out of it” [EI4].

the cost of production is so low they’re willing to take a chance and bring a hundred pups over and maybe getting ten of them alive... and they’ll still make it worth their while [EI5].

Arguably, as puppies are traded as commodities, the legal trade does not need to provide for the sentience of their ‘products’, ensuring it is easy for the legal and illegal trades to blur. As Lynch et al. (2013) argue, the harms implicit in the puppy trade can be explained by the political economic context of consumer culture.

Negative impact extends well beyond the puppies (and their mothers) in the trade; experts argued it has an effect on the state of the nation’s dogs’ health with: known diseases outbreaks, the potential introduction of new diseases, and long term behavioural and health issues resulting from inappropriate breeding, rearing, transportation and socialisation of puppies. These issues in turn impact the financial stability of the legitimate puppy market, consumers, government agencies, NGOs, and the UK public, which have to contend with the health (zoonotic diseases—see also Zucca et al 2020) and social consequences of the illegal trade, as well as try to respond to it. The involvement of OCGs in the trade presents increased and new serious consequences, including illicit gains financing other serious criminality. This is exacerbated by the increased distance between consumer and breeder. The rural isolated location of puppy farms and their flow to urban centres, often in other countries, ensures that welfare and public health issues are largely invisible to consumers (Maher and Wyatt 2019). The growth in third-party and online sales establishes further distance between consumers and the lived experiences of puppies in the trade.

Criminal actors exploit legislative loopholes, enforcement capabilities and consumer detachment to manipulate and hide behind the legal trade. As indicated above, each stage of the legal regulated trade requires paperwork, documentation and/or regulation of some sort—a license, a veterinarian check, a paperwork/microchip check at the border and more recently online in the UK. Evidence suggests paperwork can be easily forged, or fraudulent versions used to make the trade appear legal and that the puppy has the proper documentation, treatments and age to be sold. Consequently, since 2015 (UK) Border Force have recognised the possible involvement of OCGs in puppy trade networks. This has been echoed by key NGOs and transport carriers, who have reported the “sheer number of illegal puppies—one in every tenth car” and “widespread and organised criminal network and activity” (APHA 2017: no page). Nonetheless, due to the diverse nature of the illegal trade, especially embedded within the legal trade, meeting records from a 2016 RSPCA and DEFRA organised workshop identified “there are still huge unknowns in how the criminal networks operate” (APHA 2017: no page). The puppy trade, in both its legal and illegal forms, is organised, but this does not necessarily mean OCGs are involved. The next section unpacks these connections drawing on the conceptualisation of OCGs by EUROPOL.

## The role of organised crime in the trade

As indicated by the structure and actors outlined in Table 1, the puppy trade is an organised industry. The illegal aspect of the trade is structured around numerous offenders across-borders and intertwined inextricably with the legal trade. Added to this is an increasingly lucrative market, with few potential risks for offenders, leadings us to speculate that the market could be vulnerable to infiltration by OCGs.

The first factor that makes such infiltration possible is the international and national dimensions of the trade. In the UK context, there are incoming (il)legal flows from Ireland and several Eastern European countries. The extensiveness of this movement and the coordination required to successfully move live ‘contraband’ over such distances means the criminals are likely to be well-funded and well-connected. This level of sophistication makes it possible that OCGs could be undertaking at least the smuggling element of the illegal puppy trade. The extensive amount of movement within countries, again in the UK in particular, also supports this possibility. For example, puppies are moved through an organised and sophisticated distribution network across the UK:

So puppies are basically coming in from Central and Eastern Europe, but that’s your starting point, but these puppies will be distributed right across the country. The individuals involved are becoming very clever…Certainly we’re aware that some of the service stations down in Kent are being used for the transfer of puppies, not necessarily to owners… they’re effectively using a distribution network to get them across the country [EI2].

Connected to the international element is the use of ports by these criminals. Ports and border crossings are corruptible; bribes to officials create paid corridors for contraband to be moved. Corruption, and bribery specifically, is a known aspect of how OCGs operate (EUROPOL 2017; Wyatt et al. 2020).

The second factor is the blurred line between the legal pet trade to facilitate trafficking. Illegal wildlife trade research documents how organised criminals exploit regulatory loopholes and launder illegally caught animals using fake documents to trade them as captive bred (Wyatt et al. 2020). Wyatt et al. identify the intertwined relationship between legitimate formal organisations and external groups characterises much wildlife trafficking. Within the puppy trade, the link to the legitimate industry provides the necessary documents and processes to launder and exploit loopholes. The use of forged and fraudulent paperwork and bribery, and the aforementioned PETS puppy movement loophole are well documented in the puppy trade. That puppy farming in Eastern Europe is legal presents an excellent opportunity and business model to mass produce puppies that are worth over £1000 in a location that is relatively cheap (costing as little as £25 per puppy). Engagement with an organised network of legitimate and illegitimate actors allows an increase in scale and sophistication, reducing costs and risks and increasing fluidity and profits. Given the infrastructure required to breed and move puppies, linking up with established businesses provides OCGs with easy access to a low risk, high profit enterprise. The limited regulation and enforcement of the legal trade is key to the value and success of this overlap. The involvement of these various types of actors was evidenced in our stakeholder survey; respondents (n = 53) identified illegal puppy suppliers as mostly part of an organised network (43 respondents), but also opportunistic offenders (30 respondents) and part of legitimate business (20 respondents).

The profits from the illegal puppy trade are substantial and increasing, which is the third factor indicating possible involvement of OCGs. This stems from the increasing volume of consumer demand and competition for designer breeds (inflating their value), which has reportedly expanded during COVID-19 (Kale 2020), and the absence of a legitimate market to fully meet this demand. It is noteworthy that in both the expert interviews and consumer focus groups the threat of violence was reported when engaging with illegal/irresponsible sellers and breeders. The use or threat of violence is one of the main resources used by OCGs to maintain profits. Finally, the illegal puppy trade is flexible and has a complex modus operandi linked to digital systems (Stage 5—advertising). The puppy trade has changed rapidly in response to market conditions and demands (linked to consumer purchasing preferences, breeding rates, keeping up with ‘fashion’ and now with COVID-19). The supply chain mirrors that of global online retailers, whereby breeding, advertising, and selling coincides with peak purchasing months (e.g., Christmas and Spring). Online shopping also appeals to the nature of consumers: need for instant gratification, impulse buying with little research, trust in the internet as a buying tool, and willingness to buy without evidence of the conditions of the breeding or rearing. Consequently, the move to digital systems has greatly facilitated the ability of offenders to adapt and organise themselves. OCGs are known to have adapted their practices to the digital age; the puppy trade provides an ideal opportunity to use deception online, while relying on consumers to make emotion-driven purchases and the absence of adequate regulation.

The UNTOC definition of OCGs outlines the number of actors (three or more people), their longevity (over a substantial time period) and their purpose (acting to make money or other material gains). As we indicated, EUROPOL has expanded upon what they felt was a definition that did not capture important elements of the nature of OCG activities:

OCGs operate in a criminal economy dictated by the laws of supply and demand and are favoured by social tolerance for certain types of crime…individual criminals and criminal groups are flexible and quickly adapt to exploit new victims, to evade countermeasures or identify new criminal opportunities (EUROPOL 2017: no page).

Alongside this, EUROPOL point to the adoption of new technologies, in particular the expansion of online trade, document fraud, and money laundering. These factors, which shape European organised crime, are evident in the illegal puppy trade. In particular, the international and national dimensions of the trade, the link between legitimate and illegitimate trade, flexible and complex modus operandi, exploitation of consumers and vulnerable non-human animals, financial reward in the trade, use of online technology and links to document fraud and tax evasion—all of which we outlined above. Although we did not find concrete evidence or specific groups in our data, the structure and composition of the illegal puppy trade seem to lend to what EUROPOL and others know about how OCGs operate in Europe. Importantly, we need to be vigilant to the possibility as there are significant implications if OCGs are or do become actors in the illegal puppy trade, as we now discuss.

### Implications

We conclude by considering the implications associated with the involvement of OCGs in the puppy trade and responding to this element of the trade in the context of the wider political and economic climate.

#### Harms

As we noted above, there is extensive suffering and death of the puppies and breeding mothers in the trade. Their poor health and wellbeing further impact the general dog population, public health and have negative financial and social consequences that extend to the government, civil society and the public. OCG involvement in the trade will exacerbate these harms. First, the scale and severity of the abuse experienced by the dogs will escalate, alongside the increased volume of dog’s bred and traded and the development of processes aimed to maximise profits. This will increase the potential for zoonotic diseases, negative behaviour and health, and the abandonment, and euthanasia of healthy dogs. The international dimensions of the trade particularly exacerbate health concerns; the Netherlands and France have traced rabies cases back to puppies imported from non-rabies-free countries (Diamantopoulou 2019). Second, OCGs are difficult to combat. Such groups have significant resources to prevent being detected and to avoid prosecution if caught. Their financial and political power, and fluidity and sophistication would significantly change the responses and resources necessary to tackle the trade. The final concern is the consequences of the substantial profits from the illegal puppy trade supporting organised crime. Tens or hundreds of millions of pounds from the illegal sale of puppies could be entering the bank accounts of OCGs and supporting other criminal markets like drugs and weapons. Regardless of OCGs presence in the illegal puppy trade, efforts should be made to reduce this black market, but the involvement of OCGs may change what those efforts should be.

#### Regulation and enforcement

The current approach to regulating the puppy trade and the limited enforcement and resources given to this regulation do not work. Changes are required which target the modus operandi of the criminal networks. Traceability is key. Universal puppy microchipping recorded in a harmonised European Identification and Registration system to track international movement would greatly facilitate prevention, detection and control. The EU Regulation on Transmissible Animal Diseases Animal Health Law coming into force April 2021 will provide this transparency and oversight; however, it will not include the UK. Transparency and monitoring online are also central to removing the anonymity enjoyed by advertisers. This could include compulsory registration and visible tax numbers for all dog sellers (as is the case in France, CAROcat 2015) and mandatory seller verification and compliance checks by online platforms. Enhanced online traceability is part of the proposed EU Digital Service Act, which will also not apply to the UK. In the UK context, Brexit presented a unique opportunity to make enforcement robust with the possibility to change PETS, but it is not clear at the time of writing that such steps have been taken in the new legislation being debated (e.g. increasing the minimum age requirement for moving or importing dogs, decreasing the numbers permitted and enhancing rabies requirements—EFRA 2020; McCulloch 2019). Of particular concern for the UK puppy trade is that animal sentience was not included in the UK’s withdrawal bill from the EU although it is recognised in the Lisbon Treaty. While the UK Government has stated they will draft UK legislation that reflects animal sentience before the UK leaves the EU, this still has not taken place. There is the risk that the UK will be placed in a worse position as the EU improves their animal welfare legislation and tracing system and the UK is not integrated into these initiatives (i.e. the EU Action Plan against illegal trade in cats and dogs). Traceability has become even more important given COVID-19 and trade involving non-EU countries and Russia (Eurogroup for Animals 2020).

Improved monitoring and physical scrutiny (e.g. visual checks) at the ports and of breeding establishments are required to stop the spread of disease and protect public health, but also to reduce the abuse of non-human animals. Criminal networks and black markets, such as puppy trafficking, are flexible. Thus, enforcement also needs to be flexible, meaning law enforcement must have the resources necessary to be flexible and respond to the changing black markets. Law enforcement should also be given and trained to use a wider range of tools. For instance, the process for seizure of criminal assets in drug trafficking cases should be applied in cases of puppy trafficking. Lastly, puppy-trade campaigns aimed at consumers (e.g. Lucy’s Law, Pet fished) must emphasise that their purchase could be profiting organised crime.

## Conclusion

Despite growing concerns among NGOs, enforcement agencies, politicians (EFRA 2020) and the public, the illegal puppy trade is a buoyant and lucrative enterprise, multifarious in its nature, harm and the complexities it poses. There is growing evidence that legal pet trades—in wildlife and domestic animals—have opened the door to criminality and are increasingly of interest to OCGs. While the former has been addressed in academic literature, the trafficking of ‘domestic’ companion animals is largely overlooked. Likewise, within organised crime studies, non-human animals, especially domesticated animals, are mostly ignored. This paper has aimed to fill this disparity by detailing the findings of a UK multi-method qualitative study on the nature and impact of the illegal puppy trade to evaluate the role of OCGs.

The study identified a trade, international in scope, involving complicated stages, activities, and actors. The structure and actors outlined herein indicate the puppy trade is an organised industry. The illegal trade is structured around numerous offenders across-borders and intertwined inextricably with the legal trade. Added to this is the ability of criminal actors to respond fluidly to the changing market, and exploit legislative loopholes, enforcement capabilities and consumer detachment. Growing consumer demand makes the trade attractive to criminal actors, who can tap into the emotive desire for dogs. Regrettably, as their value to people grows, so too does their value for criminals and their likeliness to experience suffering and death. The material objectification inherent in the trade leaves an increasing number of dogs vulnerable to abuse and exploitation. Their suffering poses serious harm to public health and has negative financial and social consequences that extend to the government, civil society, and the public. Yet, criminal actors manipulate and hide behind the legal trade, with little risk of detection or punishment.

Although our study did not specifically identify OCGs, using the EUROPOL definition, the factors shaping European organised crime are evident in the illegal puppy trade. In particular, the international and national dimensions of the trade, the link between legitimate and illegitimate trade, flexible and complex modus operandi, exploitation of consumers and vulnerable non-human animals, financial reward in the trade, use of online technology and links to document fraud and tax evasion.

Although the European Parliament (2019) links the European illegal pet trade to serious and organised crime, we lack clarity on the involvement of OCGs in the puppy trade. Despite this uncertainty changes are needed to the regulation and enforcement of the trade, which suffers from a general lack of awareness and prioritisation, limited data collection and resources, and inadequate penalties. Current legislation does not recognise the reality of this trade, providing loopholes which are exploited by criminals. Understanding the role and modus operandi of OCGs has important implications for developing targeted prevention and detection strategies. Transparency and traceability in the origin and movement of puppies, alongside improving the physical/visual scrutiny and monitoring at borders and online, are central to such a targeted response. Arguably Brexit and COVID-19 provide significant opportunities to both reform the puppy trade and non-human (and human) animal welfare in the UK. However, at the time of writing, policy development indicates insufficient commitment and clarity for such potential to be realised. This is particularly problematic at a time whereby key EU responses to the trade are coming into effect (e.g. EU Animal Health Law, EU Digital Services Act, Bio-crime Model (Zucca et al 2020)) and there is growing recognition of the need for international collaboration and cooperation, especially in response to this emerging organised crime.

## Footnotes

1 In recognition of the debates surrounding the harms perpetuated by anthropocentric and speciesist discourse and the artificial distinction used to distinguish humans from other animals, we use the term non-human animals herein.

2 While this paper specifically refers to the puppy trade, NGO and governmental evidence suggests a growing international trade in cats and rabbits.

3 While it is beyond the scope of this article to provide a detailed discussion on the definition of OC or OCGs, further examination of the limitations and alternative definitions is provided by Wyatt et al. (2020).

4 While it is beyond the scope of this article to provide a detailed review of past literature, an annotated bibliography and literature review is available in Wyatt et al. 2017b

5 See for example, The Animal Welfare (Breeding of Dogs) (Wales) Regulations 2014 [UK] or Dog Breeding Establishment Guidelines 2018 [Ireland].

6 Balai Directive [Council Directive 92/65/EEC].

7 For the commercial movement of dogs (including a change of ownership or consignments of 5 or more pets) within the EU an Intra Trade Animal Health Certificate (ITAHC) must be completed prior to departure. For the non-commercial movement of pets (e.g. less than six companion dogs) a PET passport must be completed prior to departure. Under the NI Protocol, from 1 January 2021, regulation No 576/2013 will continue to apply to travel between NI, EU Member States and Third Countries, however Great Britain (GB) has become a ‘Part II Listed’ Third Country, for the purposes of pet travel. Dogs travelling from GB to the EU must now complete an Animal Health Certificate.

8 Further details on the specific harms in the trade are detailed in Wyatt et al. (2017b).
